# Body Fat Accumulation in Zebrafish Is Induced by a Diet Rich in Fat and Reduced by Supplementation with Green Tea Extract

**DOI:** 10.1371/journal.pone.0120142

**Published:** 2015-03-18

**Authors:** Shinichi Meguro, Takahiro Hasumura, Tadashi Hase

**Affiliations:** Biological Science Research, Kao Corporation, Haga-gun, Tochigi, Japan; Shanghai University of Traditional Chinese Medicine, CHINA

## Abstract

Fat-rich diets not only induce obesity in humans but also make animals obese. Therefore, animals that accumulate body fat in response to a high-fat diet (especially rodents) are commonly used in obesity research. The effect of dietary fat on body fat accumulation is not fully understood in zebrafish, an excellent model of vertebrate lipid metabolism. Here, we explored the effects of dietary fat and green tea extract, which has anti-obesity properties, on body fat accumulation in zebrafish. Adult zebrafish were allocated to four diet groups and over 6 weeks were fed a high-fat diet containing basal diet plus two types of fat or a low-fat diet containing basal diet plus carbohydrate or protein. Another group of adult zebrafish was fed a high-fat diet with or without 5% green tea extract supplementation. Zebrafish fed the high-fat diets had nearly twice the body fat (visceral, subcutaneous, and total fat) volume and body fat volume ratio (body fat volume/body weight) of those fed low-fat diets. There were no differences in body fat accumulation between the two high-fat groups, nor were there any differences between the two low-fat groups. Adding green tea extract to the high-fat diet significantly suppressed body weight, body fat volume, and body fat volume ratio compared with the same diet lacking green tea extract. 3-Hydroxyacyl-coenzyme A dehydrogenase and citrate synthase activity in the liver and skeletal muscle were significantly higher in fish fed the diet supplemented with green tea extract than in those fed the unsupplemented diet. Our results suggest that a diet rich in fat, instead of protein or carbohydrate, induced body fat accumulation in zebrafish with mechanisms that might be similar to those in mammals. Consequently, zebrafish might serve as a good animal model for research into obesity induced by high-fat diets.

## Introduction

Although obesity is preventable, its incidence has nearly doubled worldwide since 1980 [1]. Metabolic syndrome, which represents a cluster of conditions including high blood pressure, high blood sugar levels, excess fat around the waist, and abnormal cholesterol levels, is becoming more common as a result of the increasing prevalence of obesity [2]. Therefore, obesity is considered to be a key risk factor for noncommunicable diseases such as cardiovascular diseases, diabetes, musculoskeletal disorders, and some cancers [1].

Epidemiological studies have shown a positive relationship between dietary fat intake and obesity [3]. A high-fat (HF) diet not only induces obesity in humans but also makes laboratory animals obese [4–6]. In rodents, a positive relationship has been found between dietary fat content and body fat gain [7–10]. The C57BL/6J mouse, which has become an important model for understanding the interplay between genetic background and environmental challenges such as HF or high-calorie diets, is predisposed to the development of metabolic syndrome [11]. This model has been shown to be valid for establishing basic biochemical changes and performing preclinical studies on potentially useful drugs and functional food ingredients such as fish oil and green tea extract (GTE) [12–15].

The organs and tissues of zebrafish (*Danio rerio*) are similar to those of humans in terms of both structure and function. Consequently, the zebrafish is increasingly being used as a model of human disease because it is amenable to genetic manipulation, it breeds readily in captivity, and experimental colonies can be inexpensively maintained [16]. In addition, several studies have found that the zebrafish is an excellent model of vertebrate lipid metabolism [17, 18]. Overexpression of the endogenous melanocortin antagonist agouti-related protein and the serine/threonine protein kinase Akt1 induce adiposity in zebrafish [19, 20]. In addition, Oka et al. [21] demonstrated the usefulness of overfed zebrafish as a model of diet-induced obesity that shares common pathophysiological pathways with mammalian obesity. Indeed, Hasumura et al. [22] demonstrated the anti-adiposity effect of GTE, which has anti-obesity effects, by using this diet-induced obese zebrafish model. These reports suggest that zebrafish can be a useful animal model for obesity research. However, the effect of dietary fat on body fat accumulation in teleost fishes is not well understood.

Here, we investigated the effect of fat in the diet on body fat accumulation in zebrafish. We also examined the effect of GTE supplementation on body fat accumulation induced by a diet rich in fat, as well as on fatty acid oxidation enzyme activity in the liver and skeletal muscle of zebrafish.

## Materials and Methods

### Ethics statement

All animal experiments were carried out in strict accordance with the regulations approved by the Animal Care and Experimentation Facility Committee of Kao Corporation and with those outlined in *The Zebrafish Book* [23] and *Guide for the Care and Use of Laboratory Animals* 8th edition [24].

### Animals

Adult *D*. *rerio* were purchased from a local pet supplier (Meito Suien Co., Ltd., Remix, Nagoya, Japan). All fish were raised and maintained under a 14:10-h light:dark cycle at 28°C, and water quality conditions were maintained according to *The Zebrafish Book* [23]. The zebrafish used in each experiment described below were offspring of the purchased fish. They were from the same litter and were aged 5.5 to 7 months post-fertilization.

### Diet ingredients

The standard zebrafish chow Otohime B2 was obtained from Marubeni Nisshin Feed Co., Ltd. (Tokyo, Japan). Gluten was obtained from Wako Pure Chemical Industries, Ltd. (Osaka, Japan), and α-potato starch, corn oil, and lard were purchased from Oriental Yeast Co., Ltd. (Tokyo, Japan).

GTE was prepared and analyzed as previously described [22, 25]. The composition of catechins was measured by high-performance liquid chromatography. The total catechin content in the GTE was 79.6% to 81.8% (w/w), which comprised epigallocatechin gallate (accounting for 43.6%–44.4% of the total), epigallocatechin (20.4%–20.7%), epicatechin gallate (12.0%–12.3%), epicatechin (8.1%–8.3%), gallocatechin (6.9%–7.0%), gallocatechin gallate (4.4%), and others (3.7%–3.8%). The caffeine content was 0.0% to 0.1%.

### Experimental design

#### Experiment 1

Female adult zebrafish were weighed under anesthesia with 0.0075% (w/v) tricaine (Sigma-Aldrich, St. Louis, MO, USA) and allocated to four groups (*n* = 16 in each group) with similar body weights. Eight fish were placed in each 1.7-L tank. For 6 weeks, each group was fed one of the four experimental diets: low-fat (LF) 1, LF2, high-fat (HF) 1, or HF2 (Table 1). Each tank of eight fish received 80 mg of the experimental diet twice daily. During feeding, water inflow to the tanks was paused for 45 min and the fish were allowed to consume the diet for 30 min. On the last day of the experiment, all fish were euthanized and their body weights and body fat volumes were measured.

**Table 1:** Nutrient compositions of low-fat (LF) and high-fat (HF) diets.

Ingredient (%, w/w)	LF1	LF2	HF1	HF2
Starch	20	-	-	-
Gluten	-	20	-	-
Corn oil	-	-	20	-
Lard	-	-	-	20
Basal diet^1^	80	80	80	80
Nutrient (%)				
Carbohydrate	20	-	-	-
Protein	60	80	60	60
Fat	4	4	24	24
Ash and others	16	16	16	16
kcal/g diet^2^	4.636	4.936	5.676	5.676

^1^ Basal diet contains 40% gluten and 40% Otohime B2.

^2^ Calorific values of foods were estimated from heat of combustion; for carbohydrate, protein, and fat they were 4.20, 5.70, and 9.40 cal/g, respectively.

#### Experiment 2

Male and female adult zebrafish were weighed under anesthesia with 0.0075% (w/v) tricaine and allocated to two groups of each sex (*n* = 16 in each group) with similar body weights. Eight fish were placed in each 1.7-L tank. For 6 weeks, the two male groups were fed either the HF2 diet or HF2-GTE diet (i.e., HF2 plus 5% [w/w] GTE), as were the two female groups. Each tank of eight female fish received 80 mg of the HF2 diet or 84 mg of the HF2-GTE diet twice daily, whereas the male groups received half the amount of each diet. On the last day of the experiment, all fish were euthanized and their body weights and body fat volumes were measured.

#### Experiment 3

Female adult zebrafish (*n* = 16 in each group) with similar body weights were fed either the HF2 diet or the HF2-GTE diet over 2 weeks. On the last day of the experiment, all fish were euthanized and the liver and dorsal skeletal muscles were dissected. Liver and skeletal muscle samples were stored at −80°C until analysis of enzymatic activity.

### Measurement of food intake

Food intake in each tank was monitored for 5 days consecutively each week during the experimental period. The amount consumed was scored visually on a 10-point scale from 0 (all food left) to 1.0 (no food left) by the same person, who was blinded to the study design. The food intake of the eight fish in each tank was calculated as the amount fed multiplied by the food intake score (from 0 to 1.0). On the other 2 days a week, the fish were also fed, but food intake was not scored. Therefore, we used the average score of the 3 days prior for the first missing day and the average score of the 3 days after for the second missing day. Energy intake was calculated from the food intake and expressed as calories per fish.

### Computed tomography measurement of body fat volume

Body fat volume was measured with a 3D micro-CT scanner (Rigaku Corporation, Tokyo, Japan) as previously described [22]. The Hounsfield unit value of fat tissue was adjusted to between −350.0 and −145.0 in accordance with the manufacturer’s instructions. Measurement of body fat volume was limited to the abdominal cavity. Body fat was then divided into visceral fat and subcutaneous fat along the ribs.

### Fatty acid oxidation enzyme activity in the liver and skeletal muscle

#### 3-Hydroxyacyl-coenzyme A dehydrogenase (3-HAD) activity

The activity of 3-HAD, which is a component of mitochondrial fatty acid β-oxidation, was measured as previously described [26], with minor modifications. Briefly, frozen livers and muscles were separately thawed and homogenized in 1 ml of HES buffer (250 mM sucrose, 20 mM HEPES, 1 mM EDTA, pH 7.2) for 2 min by using a mixer mill (MM 300, Retsch GmbH, Haan, Germany) and a zirconia ball (5-mm diameter; AS ONE, Osaka, Japan). Subcellular debris was removed by centrifugation at 800 ×*g* for 5 min, and the obtained supernatant was used as the crude enzyme sample. This crude sample was diluted 1:5 with HES buffer for use in the assay. To measure enzyme activity, 100 μl of assay sample was added to 800 μl of reaction buffer (125 mM triethanolamine [pH 7.0], 0.5625 mM NADH, and 6.25 mM EDTA) in a microcuvette (light path, 1 cm; Bio-Rad, Hercules, CA, USA). The cuvette was incubated at 30°C for 5 min, and the reaction was initiated by adding 100 μl of 1 mM acetoacetyl-CoA. Activity was measured spectrophotometrically (UV-1650PC, Shimadzu, Kyoto, Japan) by observing the consumption of NADH at a wavelength of 340 nm for 5 min.

#### Citrate synthase (CS) activity

CS activity was measured as described previously [27], with minor modifications. Briefly, the crude enzyme sample described above was diluted 1:10 with 100 mM Tris-HCl solution (pH 8.0) for use in the assay. To measure enzyme activity, 100 μl of assay sample was added to 800 μl of reaction buffer (0.125 mM dithionitrobenzoic acid, 0.375 mM acetyl-coenzyme A, 125 mM Tris-HCl) in a microcuvette (light path, 1 cm). The cuvette was incubated at 30°C for 5 min, and the reaction was initiated by adding 100 μl of 5 mM oxaloacetate. Activity was measured spectrophotometrically (UV-1650PC, Shimadzu) by observing the production of thionitrobenzoic acid at 412 nm for 5 min.

### Statistical analysis

All data are reported as means ± SE. The significance of observed differences was evaluated by analysis of variance followed by application of Fisher’s partial least-squares difference multiple comparison. A difference was considered to be significant at *P*<0.05. Statistical analyses were performed with Stat-View for Windows (version 5.0; SAS Institute Inc., Cary NC, USA).

## Results

### Effect of dietary fat on body fat accumulation in zebrafish

**Fig 1:**
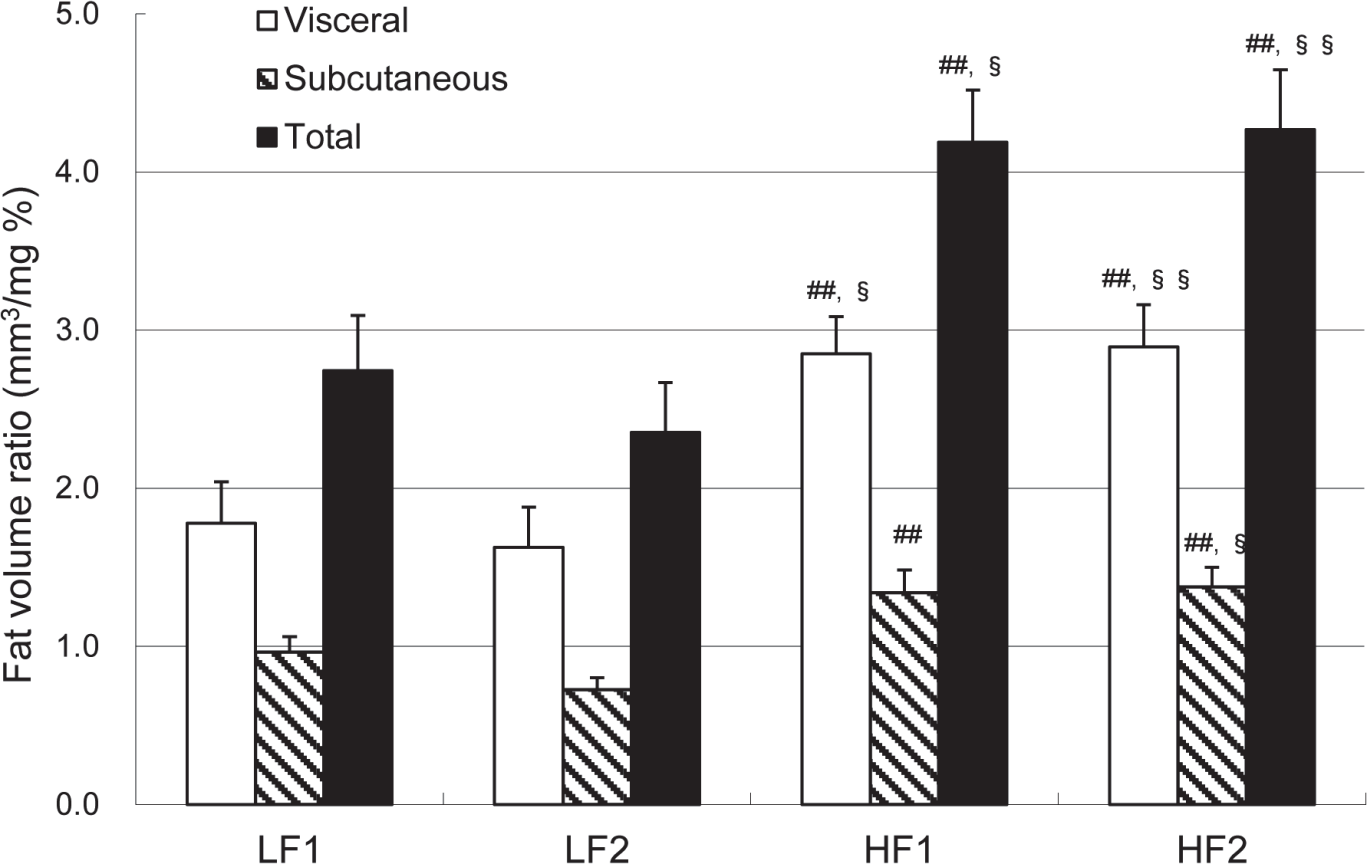
Body fat volume ratios in zebrafish fed low-fat (LF) or high-fat (HF) diets. For 6 weeks, female adult zebrafish were fed the LF1, LF2, HF1, or HF2 diet (*n* = 16 in each group). On the last day, fish were euthanized and body weights and body fat volumes were measured by using CT. Values are means ± SE. ^##^ *P* < 0.01 compared with LF1 diet. ^§^ *P* < 0.05 and ^§§^ *P* < 0.01 compared with LF2 diet.

During the experimental periods, no marked abnormalities or major differences were observed in feeding behavior among the four diet groups (i.e., LF1, LF2, HF1, and HF2). Average energy intake in the HF diet groups was about 10% to 15% greater than that in the LF diet groups, whereas no differences existed between the two LF diet groups or between the two HF diet groups (Table 2). Feed efficiency, calculated from body weight gain and average energy intake, did not differ among the four diet groups. The visceral, subcutaneous, and total body fat volumes were significantly greater in the HF diet groups than in the LF diet groups (Table 2), but there were no differences between the two LF diet groups or the two HF diet groups (despite the different fat sources of corn oil versus lard in the HF1 and HF2 diets). The visceral, subcutaneous, and total body fat volume ratios, calculated from body fat volume and body weight, were also significantly higher in the HF diet groups than in the LF diet groups, whereas no differences were observed between the two LF diet groups or the two HF diet groups (Fig. 1).

**Table 2:** Energy intake, body weight, feed efficiency, and body fat volume in zebrafish fed a low-fat (LF) or high-fat (HF) diet.

	LF1	LF2	HF1	HF2
Energy intake (kcal/fish)	3.62	3.57	3.99	4.16
Body weight (mg)				
Initial	812.2 ± 12.1	812.6 ± 11.7	812.4 ± 13.1	813.9 ± 9.3
Final	1168.7 ± 24.8	1139.7± 30.9	1193.9 ± 41.4	1222.2 ± 23.6 ^#^
Feed efficiency (mg/kcal/fish)	98.4 ± 6.4	91.6 ± 8.1	95.5 ± 9.7	98.0 ± 5.4
Body fat volume (mm^3^)				
Visceral fat	20.3 ± 2.9	18.5 ± 3.1	34.4 ± 3.7 ^##^ ^,^ ^§§^	35.3 ± 3.3 ^##^ ^,^ ^§§^
Subcutaneous fat	11.2 ± 1.2	8.3 ± 0.9	16.5 ± 2.3 ^##^ ^,^ ^§^	16.8 ± 1.6 ^##^ ^,^ ^§^
Total fat	31.5 ± 3.9	26.7 ± 3.8	50.8 ± 5.4 ^##^ ^,^ ^§§^	52.1 ± 4.7 ^##^ ^,^ ^§§^

Values are means ± SE.

^§^ *P* < 0.05

^§§^ *P* < 0.01 compared with LF1 diet.

^#^ *P* < 0.05

^##^ *P* < 0.01compared with LF2 diet.

### Effect of GTE on HF-diet-induced body fat accumulation in female and male zebrafish

**Fig 2:**
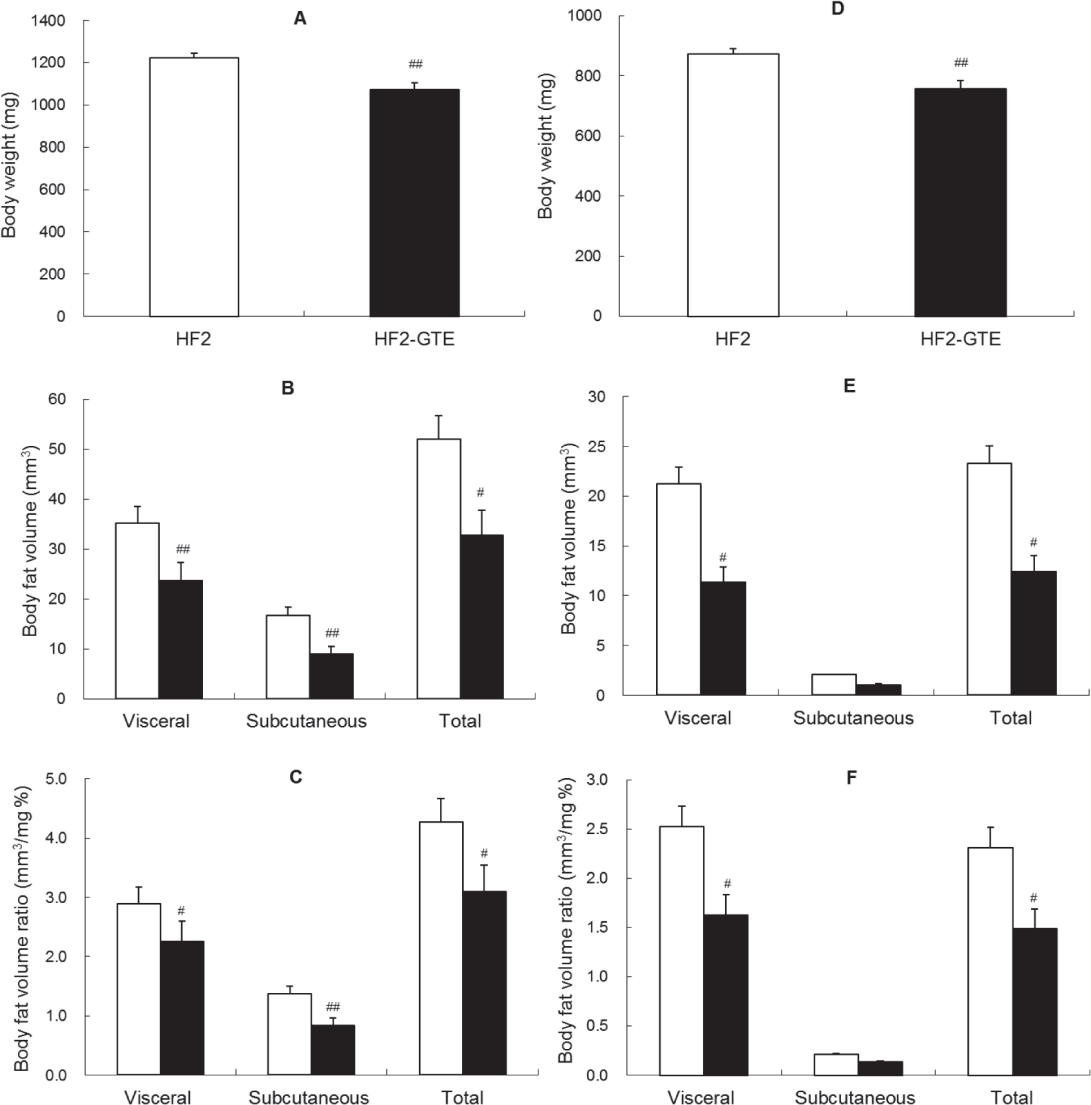
Body weight and body fat volumes in zebrafish fed green tea extract (GTE) supplemented diets. Body weight (A: female, D: male), body fat volume (B: female, E: male), and body fat volume ratio (C: female, F: male) in fish fed the HF2 diet or HF2-GTE diet for 6 weeks (*n* = 16 in each group). White and black bars show the HF2 diet and HF2-GTE diet. Values are means ± SE. ^#^ *P* < 0.05; ^##^ *P* < 0.01 vs. fish fed the HF2 diet.

The average energy intakes in the HF2 and HF2-GTE diet groups were 4.164 and 4.102 kcal/fish in females and 2.240 and 2.242 kcal/fish in males, respectively. No differences were observed between groups of the same sex. In both female and male fish, body weight was significantly lower in those fed the HF2-GTE diet than in those fed the HF2 diet (Fig. 2A, D). In females, the visceral, subcutaneous, and total body fat volumes and body fat volume ratios were significantly lower in fish fed the HF2-GTE diet than in those fed the HF2 diet (Fig. 2B, C). The same was found in male fish, except that subcutaneous body fat volume was similar between the two groups (Fig. 2E, F).

### Effect of GTE on fatty acid oxidation enzyme activity in liver and skeletal muscle

**Fig 3:**
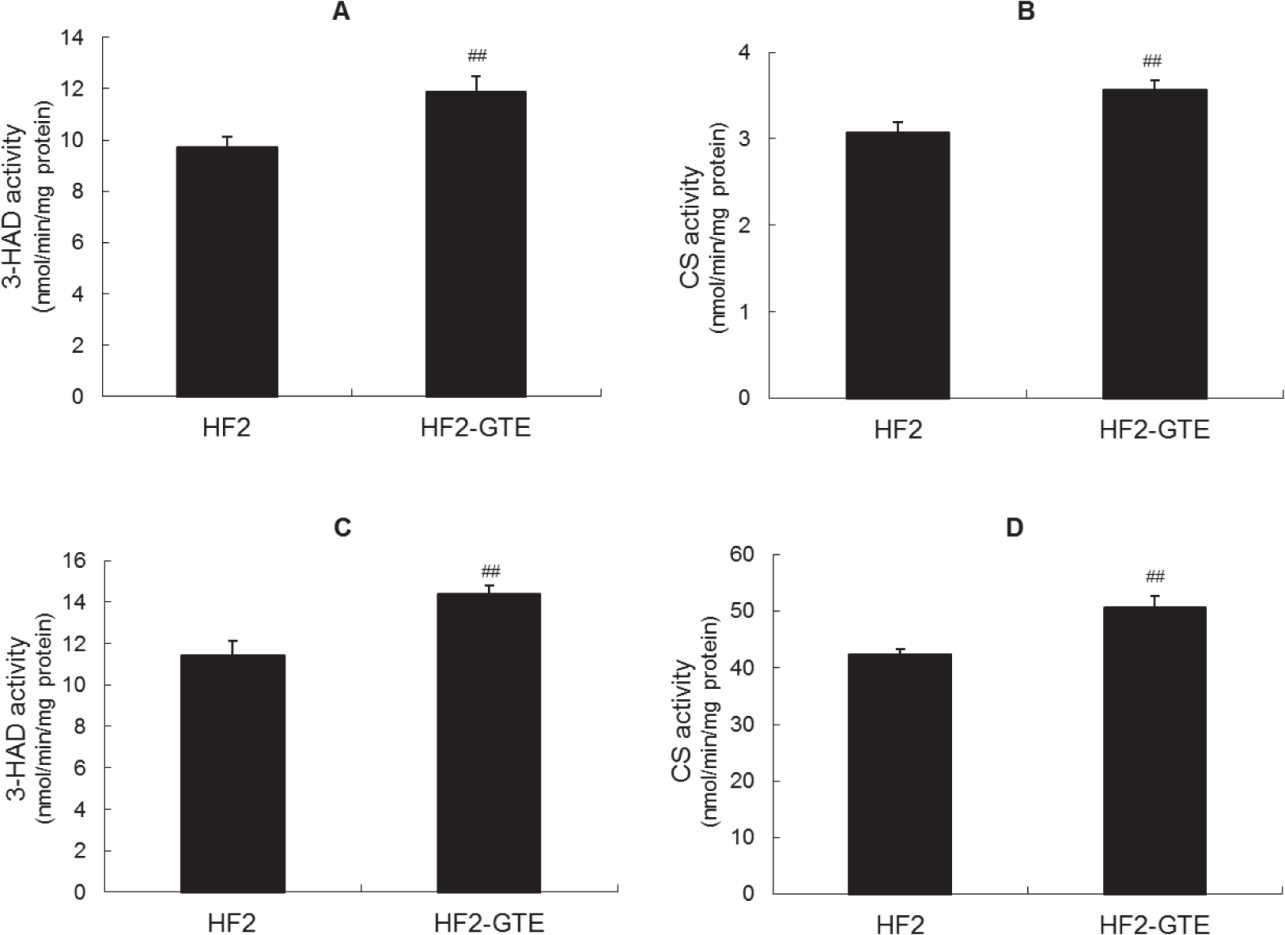
Fatty acid oxidation enzyme activity in zebrafish fed green tea extract (GTE) supplemented diets. 3-Hydroxyacyl-coenzyme A dehydrogenase (3-HAD) and citrate synthase (CS) activity in the liver and skeletal muscle of fish fed the HF2 diet or HF2-GTE diet for 2 weeks (*n* = 16 in each group). (A) 3-HAD activity in liver; (B) CS activity in liver; (C) 3-HAD activity in skeletal muscle; (D) CS activity in skeletal muscle. Values are means ± SE. ^##^ *P* < 0.01 vs. fish fed the HF2 diet.

To identify the mechanism by which GTE exerts its anti-obesity effects in zebrafish, we measured 3-HAD and CS activity levels in the liver and skeletal muscle of female adult zebrafish. 3-HAD activity in both the liver and the skeletal muscle was significantly higher in fish fed the HF2-GTE diet than in those fed the HF2 diet (Fig. 3A, C). The same was observed for CS activity in both tissues (Fig. 3B, D).

### Body fat distribution in female and male zebrafish

**Fig 4:**
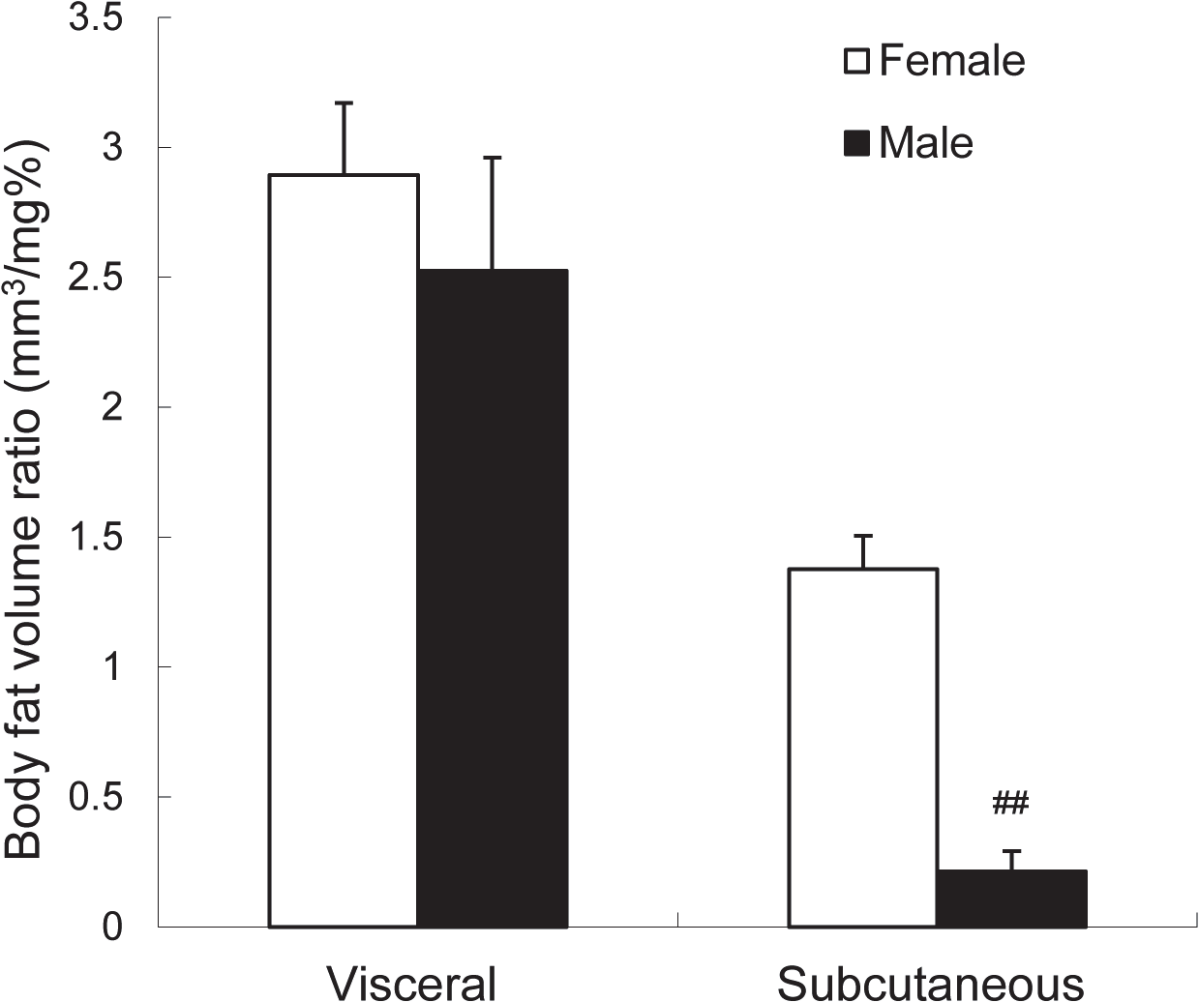
Body fat volume ratios of subcutaneous and visceral fat in female and male zebrafish. Body fat volume ratios in fish fed the HF2 diet for 6 weeks (*n* = 16 in each group). Values are means ± SE. ^##^ *P* < 0.01 vs. female zebrafish.

Body fat volume ratios in female and male fish fed the HF2 diet were 2.89 ± 0.3 and 2.53 ± 0.4 mm^3^/mg, respectively, for visceral fat and 1.38 ± 0.1 and 0.21 ± 0.1 mm^3^/mg, respectively, for subcutaneous fat. The subcutaneous fat ratio in female fish was significantly higher than that in male fish. The visceral fat ratio was similar in both sexes (Fig. 4, which presents some of the data from the HF2 diet group in Fig. 2C, F).

## Discussion

In zebrafish, we explored whether a diet rich in fat would induce the accumulation of body fat—especially visceral fat, which is a key risk factor for metabolic syndrome [28, 29]. We confirmed that increments in visceral and subcutaneous fat volume, as quantified by CT, were induced in zebrafish fed the basal diet with added fat. Matsumoto et al. [30] also reported that medaka (*Oryzias latipes*) fed a fat-rich diet exhibited fat accumulation in the liver and visceral area, as assessed histologically. In addition, many studies of salmon and trout have shown that a fat-rich diet increases the visceral fat content, as measured by lipid extraction from the visceral area [31–38]. Although these reports showed that a fat-rich diet can induce fat accumulation in the internal organs, the visceral and subcutaneous fat contents have not been analyzed quantitatively. Therefore, to our knowledge, this is the first quantitative study to show that a diet rich in fat induces body fat accumulation, in both visceral and subcutaneous areas, in a teleost fish. Zebrafish might therefore also be a good model for simply and quantitatively analyzing body fat area.

Zebrafish fed the HF diet had greater body fat volumes and body fat volume ratios than those fed the LF diet, whereas no differences were observed in feed efficiency between the two diet groups. Petro et al. [39] showed that body fat ratio was significantly greater in B6 mice fed a fat-rich diet than in those fed a low-fat diet, despite a lack of differences in feed efficiency and energy intake between the diet groups; they concluded that fat, not energy intake, is the crucial stimulus for body fat accumulation in the B6 mouse. These data indicate that the responses of body fat accumulation to dietary fat intake are parallel between zebrafish and B6 mice. On the other hand, body fat volumes and body fat volume ratios did not differ between zebrafish fed corn oil (HF1) or lard (HF2) as the fat source. Likewise, previous studies have found that body fat accumulation was affected by the amount of dietary fat but not the type of dietary fat (i.e., lard or corn oil) in Wistar rats [40] and spayed dogs [41]. These results suggest that the biochemical pathway transforming dietary fat to body fat in zebrafish might be similar to that in mammals.

We found here that a diet rich in fat, instead of carbohydrate or protein, induced body fat accumulation effectively in zebrafish; this is similar to the case in mammals [4–10]. Many kinds of ingredients, such as n-3 polyunsaturated fatty acids, soy protein, dietary fiber, and GTE, are contained in the human diet. However, the function and precise mechanisms underlying body fat accumulation have not been well defined. Compared with rodents, the zebrafish is amenable to genetic manipulation and breeds readily in captivity; moreover, experimental colonies can be inexpensively maintained [16]. Zebrafish might therefore be an efficient and useful model for investigating the functions and mechanisms of dietary ingredients in body fat accumulation.

Supplementation of the HF diet with GTE significantly suppressed body weight, body fat volume, and body fat volume ratio in male and female zebrafish as compared with those in fish fed the same diet lacking GTE. Numerous studies of male and female rodents have shown that body weight gain and body fat accumulation induced by a fat-rich diet are suppressed by dietary GTE supplementation [14, 15, 42–45]. Therefore, our results indicate that the effect of GTE on HF-diet-induced body fat accumulation in zebrafish may be similar to that seen in rodents.

3-HAD and CS activity levels in the liver were significantly higher in zebrafish fed the GTE-supplemented HF diet than in those fed the same diet without GTE. In mice, GTE supplementation of a fat-rich diet increases mRNA expression of medium-chain acyl-coenzyme A dehydrogenase (an enzyme involved in mitochondrial fatty acid β-oxidation) and β-oxidation activity in the liver; these effects are accompanied by the suppression of body fat accumulation [15, 42]. The authors of these mouse studies concluded that stimulation of hepatic lipid metabolism might be one of the molecular mechanisms underlying the anti-obesity effects of GTE. 3-HAD is involved in fatty acid metabolic processes that catalyze the third step of mitochondrial fatty acid β-oxidation [46]. CS catalyzes the condensation reaction of acetyl-coenzyme A and oxaloacetate to form citrate, and it is commonly used as a quantitative enzyme marker for the presence of intact mitochondria [47]. These findings suggest that the effect of GTE on hepatic mitochondrial fatty acid β-oxidation in zebrafish is similar to that in rodents.

3-HAD and CS activity levels were also significantly increased in the skeletal muscle of zebrafish fed the GTE-supplemented diet. In rodents, β-oxidation activity in skeletal muscle is greater in exercised mice fed a GTE-supplemented diet than in those fed an unsupplemented diet, but this increase is not observable in sedentary mice [42, 48]. Our zebrafish did not experience a special exercise load during the experimental periods, but the water inflow in the tanks was about 5 to 10 ml/s. Thus, the skeletal muscles of the zebrafish were continuously “exercised” over the course of the experiment.

Together, these findings suggest that the degree of activation of hepatic and skeletal muscle fatty acid β-oxidation by GTE ingestion may be comparable in zebrafish and rodents. This activation may be one molecular mechanism underlying the suppression of body fat accumulation induced by a fat-rich diet. Further investigations are needed to elucidate the precise mechanism.

Here, we showed that the subcutaneous fat ratio was significantly higher in female fish than in male fish, but the visceral fat ratio was similar in both sexes. The mechanism underlying this sex difference in body fat distribution in zebrafish remains unclear. In a human study, Westerbacka et al. [49] reported a doubling in volume of subcutaneous fat in women compared with that in men, but no difference was observed in visceral fat volume between women and men of similar age and body weight. Indeed, this phenomenon has been reported in numerous human studies [50–54]. Therefore, our findings raise the possibility that the body fat distributions in male and female zebrafish are similar to those in humans. Further studies are needed to understand the sex differences in body fat distribution in zebrafish.

## Conclusions

We showed here that a diet rich in fat, instead of protein or carbohydrate, as the macronutrient induced body fat accumulation in both visceral and subcutaneous areas in zebrafish. In addition, there were no differences in results between the use of lard or corn oil as the fat source in the HF diet. Adding GTE to the HF diet suppressed body fat accumulation and was accompanied by increased hepatic and skeletal muscle fatty acid oxidation enzyme activity. Moreover, a sex difference in body fat distribution was identified in the zebrafish. All of these results are similar to those found in mammals, suggesting that zebrafish can serve as a suitable animal model in research into obesity induced by a diet rich in fat.
